# Prostate volume and implant configuration during 48 hours of temporary prostate brachytherapy: limited effect of oedema

**DOI:** 10.1186/s13014-014-0272-9

**Published:** 2014-12-11

**Authors:** Anna M Dinkla, Bradley R Pieters, Kees Koedooder, Niek van Wieringen, Rob van der Laarse, Arjan Bel

**Affiliations:** Department of Radiation Oncology, Academic Medical Center, Meibergdreef 9, 1105 AZ Amsterdam, the Netherlands

**Keywords:** Prostate cancer, Brachytherapy, HDR, PDR, Catheter displacement, Oedema

## Abstract

**Background:**

In pulsed-dose rate prostate brachytherapy the dose is delivered during 48 hours after implantation, making the treatment sensitive to oedematic effects possibly affecting dose delivery. The aim was to study changes in prostate volume during treatment by analysing catheter configurations on three subsequent scans.

**Methods:**

Prostate expansion was determined for 19 patients from the change in spatial distribution of the implanted catheters, using three CT-scans: a planning CT (CT1) and two CTs after 24 and 48 hours (CT2, CT3). An additional 4 patients only received one repeat CT (after 24 hours). The mean radial distance (MRD) of all dwell positions to the geometric centre of all dwell positions used was calculated to evaluate volume changes. From three implanted markers changes in inter-marker distances were assessed. The relative shifts of all dwell positions were determined using catheter- and marker-based registrations. Wilcoxon signed-rank tests were performed to compare the results from the different time points.

**Results:**

The MRDs measured on the two repeat CTs were significantly different from CT1. The mean prostate volume change derived from the difference in MRD was +4.3% (range −9.3% to +15.6%) for CT1-CT2 (p < .05) and +4.4% (range −7.5% to +16.3%) for CT1-CT3 (p < .05). These values represented a mean increase of 1.2 cm^3^ in the first 24 hours and 1.5 cm^3^ in the subsequent 24 hours. There was no clear sign of prostate expansion from the change in inter-marker distance (CT1-CT2: 0.2 ± 1.8 mm; CT1-CT3: 0.6 ± 2.2 mm). Catheter configuration remained stable; shifts in catheter positions were largest in the C-C direction: 0 ± 1.8 mm for CT1-CT2 and 0 ± 1.4 mm for CT2-CT3.

**Conclusions:**

The volume changes derived from catheter displacements were small and therefore considered clinically insignificant. Implant configuration remains stable during 2 days of treatment, confirming the safety of this technique.

## Background

High-dose rate (HDR) brachytherapy combined with external beam radiotherapy (EBRT) is an effective method to achieve dose escalation in the treatment of localised prostate cancer. It results in increased biochemical relapse-free survival and reduced acute morbidity, compared with EBRT alone [1]. An alternative to HDR is pulsed-dose rate (PDR) brachytherapy, which also exploits the characteristics of local treatment to achieve a conformal high dose to the prostate [2-4]. The accuracy of dose delivery is an important part of a brachytherapy treatment. Inconsistencies or changes in patient and implant geometry between treatment planning and treatment delivery should be well documented and eliminated if possible [5].

In low-dose rate (LDR) permanent brachytherapy prostate swelling and accompanying seed movement caused by oedema plays a major role in the dosimetry. Authors have measured 20-30% [6-8] up to 50% increase in prostate volume [9]. Oedema arises in the first 24 hours after implantation after which it slowly resolves [8,9]. If an HDR treatment is delivered in multiple fractions, volume changes may occur between these fractions. A PDR prostate treatment is usually delivered in two days [2,3]. Therefore, PDR and fractionated HDR treatments are potentially sensitive to the effects of oedema. Preplanning is done intraoperatively, but the final dose distribution is planned a few hours after implantation. Volume changes during implantation therefore do not affect treatment, but volume changes during the treatment can affect dose delivery.

In HDR treatments, the dose is generally assumed to be minimally affected by volume changes because the fraction dose can be delivered in 15 to 20 minutes after treatment planning [10-12]. Applying more fractions with the same implant, increases the risk of changes in dosimetry. Data on prostate volume changes in the first 48 hours after implantation for HDR or PDR brachytherapy that support this assumption are sparse. One study reported data of only 4 patients [13]. Another study found a large spread in prostate volume changes one week after HDR treatment, using CT-based prostate delineations with 5 mm slices [14]. Martinez *et al.* observed the largest volume change in the first two scans and negligible change during the next 32 hours of HDR treatment [12]. Their measurements were based on delineations in axial transrectal ultrasound (TRUS) images. CT- and TRUS-based delineations however suffer from contouring uncertainty of around 2 mm [15]. Kim *et al.* found a mean volume increase of 8% 20 hours after implantation using catheter positions from 3 axial CT slices [16]. Finally, Kovalchuk *et al.* measured in 24 patients the prostate volume change between the first two fractions (separated by >6 hours) based on CT delineations, which resulted in a mean absolute change in inter-fractional volume change of 3.9 cm^3^ (range −17.7 cm^3^ to 17.1 cm^3^) [17]. The main goal of their study was to measure inter-fractional needle displacement, which is another factor that can affect dose coverage. Before delivery of each fraction, they performed needle adjustment to maintain target coverage. Catheter displacement, usually in craniocaudal direction, is commonly observed [18,19]. Huang *et al.* found that 30% of the catheters needed adjustment to prevent an 8.4% (SD 9.4%) decrease in D_90%_ [18]. Whitaker *et al.* found a median measured displacement of 7.5 mm, with shifts greater than or equal to 5 mm occurring in 67% of their implants, which was corrected for by advancing the source further into the catheters [19]. Milickovic *et al.* reported (in all but one case) a maximum needle movement of less than 1.5 mm [20], which led to a limited decrease in target coverage. At our institute, self-anchoring catheters are used to prevent large displacements.

For the PDR prostate patients, the aim was to implant a minimum number of 12 flexible plastic catheters for the 48 hour pulsed treatment. A previously published study on dose variation during these 48 hours showed only a small decrease in target coverage as compared to the planning situation: the mean difference in V_100%_ between the planning CT and the scan acquired at the end of the treatment was only −2.3% with a largest decrease of 10% [21]. Furthermore, no significant changes in absolute volume of the delineated prostates were found. However, prostate delineations suffer from considerable contouring uncertainty, especially with CT [15,22]. For treatment planning, both CT, MRI and TRUS can be used [23-25]. The main advantage of MRI is improved soft-tissue contrast for prostate boundary detection. With TRUS-based planning, moving the patient and catheter displacements are minimized [23,24]. CT is widely available and offers clear visibility of the catheters. We therefore use the configuration of the catheters measured on CT as a reliable surrogate for volumetric changes. The use of self-anchoring catheters is to ensure positional stability in the craniocaudal direction. Since the reports on the occurrence of oedema during HDR treatment vary [12-14,16] and no studies have been done for PDR treatments using self-anchoring catheters, our goal was to study volume changes of the prostate during PDR brachytherapy by analysing catheter configurations of subsequent scans acquired throughout the treatment.

## Methods

### Patients and treatment

Thirty-one prostate cancer patients have been included in a study to assess the usability of self-anchoring catheters, of which 23 were eligible for this study. This group of intermediate- and high-risk patients was treated with EBRT (23×2 Gy), followed by a PDR boost of 24.96 Gy [2,26]. The total PDR treatment consisted of 24 pulses, 2.2 hours apart.

**Figure 1:**
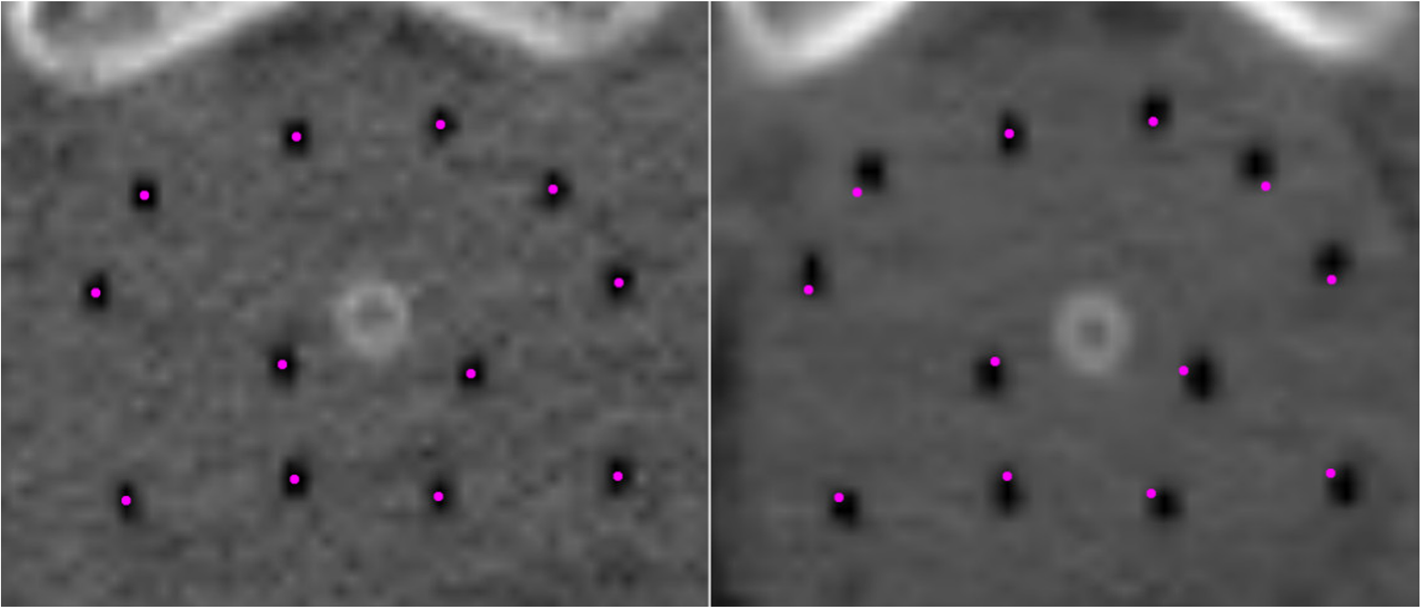
**Example of a transversal prostate image after implantation (CT1, left) and after 48 hours (CT3, right).** With the purple dots on the left image the centre of the catheters are demarcated. A small radial expansion of the catheters can be observed on the right image, illustrated by the projection of the same dots on this image. The dots were projected by taking the same slice and overlaying the two slices by matching the urethra and catheters as well as possible.

Catheter implantation was performed one week after the end of EBRT according to a pre-plan based on ultrasound images. For all patients 12 flexible plastic 6F self-anchoring catheters were implanted, except for 3 patients, for which 11 catheters were used. The catheters were placed on the periphery of the prostate to achieve a high peripheral dose (an example of the implant geometry can be seen in Figure 1). The number of catheters was independent of prostate volume, which covered a wide range (21 cm^3^ to 56 cm^3^). The implantation procedure and treatment planning were described previously [22,26]. No additional fixation of the catheters to the perineum was applied. During implantation three fiducial markers were placed in the prostate, two at the base and one at the apex, to help with the delineation of these structures on the CT scans. Placement of the markers was done under ultrasound guidance. The size of the cylindrical markers was 1 mm in diameter with 5 mm length.

The patient was lying in bed throughout the treatment. To acquire the follow-up CTs, the patients’ bed was transported from the treatment room to the CT room. There the patient was lifted on the scan table with care to avoid implant displacement. Three CT scans were made for this study. The first CT, which was used for planning, was acquired after implantation (CT1, after 1–2 hours), the second CT after 24 hours (CT2) and the third CT was made after 48 hours, right before the removal of the catheters (CT3). All scans were acquired with a resolution of 1 by 1 mm^2^ pixels and 2 mm slice thickness. For the first eight patients that received implantation with self-anchoring catheters, metal wires had been inserted in the catheters, which obscured the visibility of the implanted markers. For this reason these patients were excluded from this analysis. Of the 23 evaluable patients, 4 did not receive a third scan.

The catheters were reconstructed manually on all scans in Oncentra Brachy treatment planning system (Nucletron, Veenendaal, the Netherlands) by an experienced brachytherapist. Catheter reconstruction was based on defining 4 to 6 catheter describing points. The same person also manually defined the position of the markers on all scans.

### Measurement of prostate expansion

Prostate expansion was assessed from the dwell positions coordinates within the catheters reconstructed on CT1, CT2 and CT3. The coordinates of dwell positions inside the catheters are automatically defined by the software, spaced 5 mm apart. These coordinates were used as a spatial representation of the catheters, because of the one-to-one correspondence between these points on subsequent scans. All dwell positions inside the prostate plus a 5 mm margin were used. Then all coordinates of these dwell positions were captured scans and the centre of mass was calculated by taking the mean x and z coordinates (x_COM_, z_COM_) where x represents the left-right (L-R) direction and z the dorso-ventral (D-V) direction. We excluded the y (caudo-cranial, C-C) direction because any displacement of dwell positions in this direction represents a catheter shift unrelated to changes in volume. This is unlike in permanent seed implantation, where the seed can move in three directions. Then movement either in cranial or caudal direction can represent prostate expansion along this dimension [9].

Subsequently, from the absolute distance between the centre of mass and every dwell position the mean radial distance (MRD) was calculated by: $MRD=\frac{1}{n}{\displaystyle \sum_i^n\sqrt{{\left({x}_i-{x}_{COM}\right)}^2+{\left({z}_i-{z}_{COM}\right)}^2}}$, with n the number of dwell positions. This calculation was based on the method of Waterman *et al.* who assessed volume changes during permanent implant brachytherapy from seed positions [9]. Waterman *et al.* calculated the displacements in all three directions, whereas we excluded the C-C direction, as was also done by Kim *et al.* [16]. For every patient, MRDs were calculated for all three time points, represented by the three different CT scans. The relative volume change ΔV(%) was estimated from the relative change in MRD e.g. between CT1 and CT2 according to the method described by Kim *et al.* [16]: $\varDelta V\left(\%\right)=100*\left(\frac{MR{D_2}^3-MR{D_1}^3}{MR{D_1}^3}\right)$. We estimate the uncertainty in the MRD as follows by first choosing σ = 1 mm for the uncertainty of the distance between a dwell position (x_i_,z_i_) and the centre of mass (x_com_,z_com_). The estimated standard uncertainty in the MRD depends on the number of dwell positions: ${\sigma}_{MRD}=\frac{\sigma }{\surd n}$. The number of dwells is on average 80, making *σ*_*MRD*_ = 1 mm/√80 = 0.1 mm. The propagated uncertainty for the relative volume change DV(%) is calculated according to: ${\sigma}_{DV\%}=100\%\times \sqrt{3\left(\frac{\sigma_{MR{D}_1}^2}{MR{D}_1^2}+\frac{\sigma_{MR{D}_2}^2}{MR{D}_2^2}\right)}$ = 2% (1 SD). For an MRD_1_ ≈ MRD_2_ of 15 mm the resulting *σ*_*DV* %_ is 3% (1SD).

### Inter-marker distances

An increase in inter-marker distance between the different scans indicates the possible presence of oedema. Coordinates of the three implanted markers were recorded for all CT scans. For each scan, the distance between the three individual markers was calculated. The three marker pairs resulted in 3 distances per scan. Since each prostate contained only 3 markers, we did not evaluate the change per patient. The mean, SD and box-plots of the change in inter-marker distance (absolute and in the three separate directions) were calculated for all marker pairs combined.

### Dwell position displacement: catheter-based registration

A registration of the catheters reconstructed on subsequent scans was performed to evaluate changes in the catheter configuration. Also here the catheters were represented by all dwell positions inside the prostate. Displacement of the catheters was measured after aligning the catheters from subsequent scans, using a rigid registration of the dwell positions. The corresponding points were matched using the iterative closest point (ICP) algorithm [27] as implemented in Matlab (Mathworks, Natick, Massachusetts). ICP takes two point clouds as input and returns the rigid transformation (rotation matrix and translation vector) that best aligns the point clouds. ICP minimizes the sum of square errors, which is the sum of squared distances between matched paired points.

This ICP registration of reconstructed catheters was applied to test if the catheter configuration remained stable, i.e. whether deformation of the implant had occurred. Measured shifts between CT1-CT2 represented implant changes in the first 24 hours after implantation. The shifts between CT2-CT3 represented changes between 24 and 48 hours. The total shift between CT1-CT3 was also measured.

To estimate the accuracy of the ICP-based registration, two CT scans of a fixed 5-catheter configuration inside a phantom were acquired to register the reconstructed catheters. The phantom was not rotated or shifted but a second scan was acquired. The catheters on the two scans were reconstructed by the same person. Five feature landmarks inside the phantom were used as marker surrogates.

### Dwell position displacement: marker-based registration

The implanted markers were also used for implant displacement evaluation, since the catheter-based registration described above is not able to detect entire implant shifts. For this purpose, a marker-based registration was performed. Because only three markers were implanted, a quaternion-based method implemented in Matlab was used for this marker-based registration [28], finding the rotation and translation that best maps both sets of marker coordinates in a least squares sense. This rotation and translation was then applied to the catheters to calculate the dwell position shifts.

The same phantom with fixed catheter configuration that was used to determine the accuracy of the catheter-based registration was also used to estimate the accuracy of the marker-based method.

The marker coordinates have an uncertainty related to the voxel size. An uncertainty analysis was performed to quantify the effect of this uncertainty on the accuracy of marker-based registration of the catheters. A random noise term was added to the marker coordinates of ten patients. The coordinates were displaced 100 times with normally distributed deviations with SDs of 0.5 mm in the D-V and L-R direction and 1 mm SD in the C-C direction. These coordinates were registered as above and these marker-based registrations were applied on the dwell positions. The mean of all relative displacements resulted in the uncertainty that can be attributed to the uncertainty in marker coordinates.

### Statistical analysis

The differences in MRDs of subsequent scans as well as the prostate volumes resulting from these differences were tested for statistical significance using a Wilcoxon signed-rank test. *P*-values below 0.05 were considered statistically significant. The inter-marker distances measured on the different scans were also compared using the Wilcoxon signed-rank test.

Of the calculated dwell position shifts from the catheter- and marker-based registrations we calculated for each patient the mean (δ) ± 1 standard deviation (SD), denoting the random variation per patient (σ_pat_). The overall mean Δ and the mean standard deviation (Σ) were calculated. The root-mean-square (RMS) of the σ_pat_ from the 23 patients was calculated to estimate σ, the overall random variation over all patients. We created box-plots containing the interquartile range, median and minimum and maximum values over all dwell positions and patients for CT1-CT2 (23 patients), CT2-CT3 and CT1-CT3 (19 patients). Absolute distances, as well as shifts in all three directions were assessed.

## Results

### Measurement of prostate expansion

**Figure 2:**
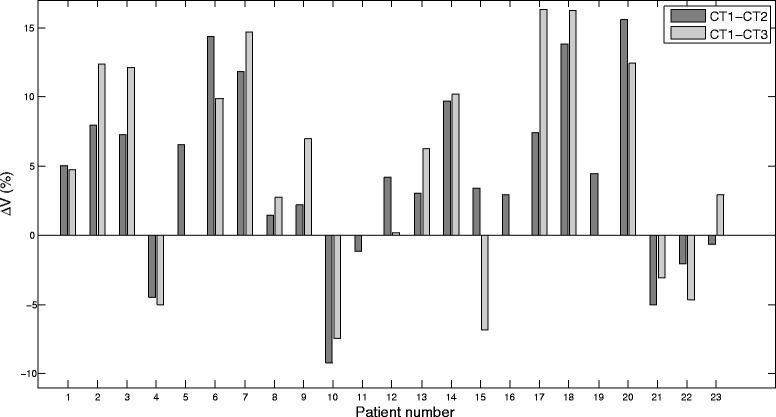
**Relative volume change (%) between CT1-CT2 and between CT1-CT3.** For patient 5, 11, 16 and 19, no CT3 was available. CT2-CT3 was omitted, since this information can easily be extrapolated from the other two bars.

The MRDs resulted in a statistically significant difference for MRD_1_-MRD_2_ and MRD_1_-MRD_3_ (p < .05). The relative volume change derived from the change in MRD was +4.3% ± 6.4% (range −9.3% to +15.6%) between CT1-CT2 (Figure 2), which was a statistically significant increase (p < .05). Although statistically significant, this represented a limited mean volume difference of 1.2 cm^3^ ± 2.3 cm^3^ (range −5.2 cm^3^ to +5 cm^3^). Taking the absolute value, the volume change was 6.2% ± 4.4%. Only for 4 (of 23) patients an increase larger than 10% was observed. For CT1-CT3, mean volume difference was +4.4% ± 7.5% (range −7.5% to +16.3%) (p < .05) (Figure 2). Between CT2-CT3, this was +0.6% ± 3.7% (range −9.9% to +8.3%) (p > .05). This represented a mean volume difference of 1.5 cm^3^ ± 2.8 cm^3^ (range −4.2 cm^3^ to 6.2 cm^3^). In absolute terms, the mean volume change was 6.7% ± 5.4%. For CT2-CT3 it was 2.7% ± 2.6%.

### Inter-marker distances

**Figure 3:**
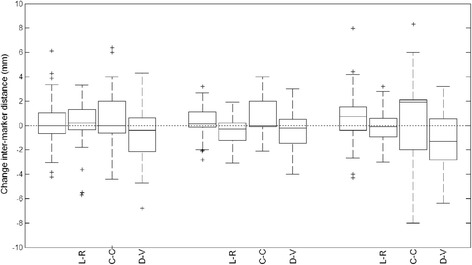
**Boxplots of the change in inter-marker distance.** For CT1-CT2, CT2-CT3 and CT1-CT3 the differences in absolute vector length are shown, as well as for the three separate directions (L-R, C-C and D-V). The dashed horizontal line represents the line of no change in distance between marker pairs. The boxes itself represent the first and third quartile (lower and upper edge), where the horizontal line inside the box represents the second quartile, i.e. the median. The crosses represent outliers.

The mean change in inter-marker distance was small in all three directions (Figure 3). Mean absolute difference was 0.2 ± 1.8 mm (1 SD) between CT1-CT2 and 0.6 ± 2.2 mm between CT1-CT3. For CT2-CT3, this was 0.4 ± 1.1 mm. Largest changes were observed in the C-C direction, with an average increase of 0.6 ± 3.0 mm between CT1-CT3.

The relative change in absolute distance between two marker pairs was 1.1% ± 7.6% (mean ± 1 SD) between CT1-CT2 (p > .05), 2.8% ± 9.5% between CT1-CT3 (p < .05) and 1.5% ± 4.9% between CT2-CT3 (p < .05). So there was a systematic increase in inter-marker distance of 1.1%-2.8% with a random difference of around 5%-10%.

### Dwell position displacement: catheter-based registration

ICP was first applied to match the catheters inside the phantom. This catheter registration resulted in a mean difference between dwell positions of 0.26 ± 0.1 mm (1 SD).

**Figure 4:**
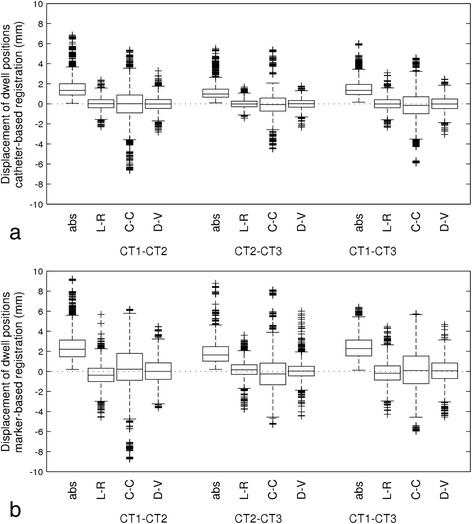
**Boxplots of displacements of the dwell positions from catheter-based registration (a) and from marker-based registration (b).** The shifts are shown as absolute vector lengths (abs) and for the three separate directions (L-R, C-C and D-V). For C-C, a positive value indicates a caudal shift; for D-V, a positive value indicates a ventral shift. The dotted horizontal line represents no displacement. The crosses represent outliers.

The relative displacement measured by catheter-based registration averaged over all dwell positions inside the catheters of all patients was 1.7 ± 1.2 mm for CT1-CT2 and 1.2 ± 0.9 mm for CT2-CT3. For CT1-CT3 this was 1.6 ± 1 mm. Shifts in the L-R and D-V directions were small. Catheter shifts in C-C direction were the largest source of dwell position displacement (Figure 4a).

In Table 1 the mean dwell position shifts are shown, averaged over all patients. The largest mean systematic shift was −0.22 ± 0.2 mm between CT1-CT3 for the C-C direction. Random variation, represented by the RMS of all standard deviations from all patients was in most cases smaller than 1 mm and largest in the C-C direction.

**Table 1:** **Residual distance between dwell positions after catheter-based and marker-based registration, averaged over all patients**

		**Catheter-based registration**			**Marker-based registration**		
		**Mean (mm)**	**1 SD (mm)**	**RMS of σ**_**pat**_	**Mean (mm)**	**1 SD (mm)**	**RMS of σ**_**pat**_
		**Δ**	**Σ**	**σ**	**Δ**	**Σ**	**σ**
CT1-CT2	Absolute	1.67	0.58	1.07	2.46	0.70	1.28
	L-R	0.00	0.06	0.64	−0.35	0.45	1.01
	C-C*	−0.11	0.19	1.72	0.22	1.44	1.83
	D-V**	−0.01	0.09	0.75	0.01	0.62	1.05
CT1-CT3	Absolute	1.63	0.47	0.94	2.39	0.70	0.95
	L-R	0.01	0.04	0.67	−0.15	0.65	0.97
	C-C*	−0.22	0.20	1.61	−0.05	1.20	1.63
	D-V**	−0.02	0.06	0.76	0.02	0.71	1.12
CT2-CT3	Absolute	1.21	0.37	0.83	1.91	0.74	1.02
	L-R	0.00	0.02	0.46	0.17	0.45	0.72
	C-C*	−0.05	0.46	1.27	−0.19	1.18	1.46
	D-V**	−0.01	0.04	0.53	0.05	0.49	0.87

Δ = Overall mean (mean of all δ).

Σ = Standard deviation of the means.

σ = Root-mean-square, quadratic mean of all standard deviations from all patients.

Data shown in absolute shifts and in the 3 separate directions.

*Positive values in C-C indicate a caudal shift.

**Positive values in D-V indicate a ventral shift.

### Dwell position displacement: marker-based registration

The registration of the markers coordinates inside the phantom on two scans resulted in a residual difference in marker position after registration of 0.1 ± 0.06 mm (1 SD). The difference in the dwell positions of the catheters that were subsequently matched according to the marker-based registration amounted to 0.7 mm ± 0.2 mm (1 SD). The accuracy of registration was therefore better than the catheter and marker position accuracy.

A separate analysis was performed to quantify the uncertainty of the measured catheter displacement due to the uncertainty in marker position. This resulted in an uncertainty of −0.01 ± 1.1 mm in L-R direction, 0.03 ± 1.4 mm in the C-C direction and 0.02 ± 1.1 mm in D-V direction.

The relative displacement of all dwell positions measured by marker-based registration (mean ± 1 SD) was 2.5 ± 1.5 mm for CT1-CT2 and 1.9 ± 1.3 mm for CT2-CT3 (Figure 4b). For CT1-CT3 this was 2.4 ± 1.2 mm.

In Table 1 the mean dwell position shifts are shown, averaged over all patients. The largest mean systematic shift was −0.35 ± 0.45 mm (1 SD) between CT1-CT2 for the L-R direction. Random variation, represented by the RMS of all standard deviations from all patients was around 1 mm and up to 1.8 mm in the y (C-C) direction.

## Discussion

This study revealed limited effect of oedema during 48 hours of temporary prostate brachytherapy, with a maximum increase in prostate volume of 16.3%. We used catheter positions to quantify volume changes to eliminate the possible effect of delineation uncertainties. The catheters are clearly visible, whereas prostate gland contour delineations on CT are subject to variation [15,22]. The change in mean radial distance (MRD) between the dwell positions and the centre of mass of the implant resulted in a mean relative volume change of +4.3% for the first 24 hours and +4.4% for the first 48 hours. With these results the safety of these techniques can be confirmed. Our data supports the conclusion that with PDR and HDR the uncertainties related to prostate volume changes are small [10,11]. If there was any oedematic effect from catheter insertion inside the prostate, it took place in the first 1.5 hours after implantation.

The mean volume increase in the first 24 hours was smaller than the 7.8% increase found by Kim *et al*. measured on CT after 20 hours [16]. They considered this clinically insignificant, due to the small change in prostate radius and the limited reduction in target coverage of around 3%. From their data no relationship between volume increase (or decrease) and target coverage decrease (or increase) could be established. An earlier dosimetric analysis of our own patient group showed only a small decrease in target coverage [21]. Only one patient showed a large decrease of 7% in V_100%_ on the second CT-scan and 10% on the third CT-scan. Kim *et al.* evaluated CT-based catheter positions in the L-R and D-V directions, but looked at the catheter coordinates in just three axial planes.

The measured volume changes were much smaller than those reported in LDR series, where expansions of around 30% was measured [6-8]. In LDR treatments, more needles are usually implanted and the pre-implant volume is usually also considered. Furthermore, an LDR treatment has a much longer duration. Waterman *et al*. found a mean increase of even 50% using the mean radial distance of seeds to the geometric centre of the implant [9]. In our series, some individual cases showed a volumetric increase of more than 10%, derived from the MRD. The largest increase in MRD was 0.8 mm, representing expansions in diameter of 1.6 mm, which could be considered as a substantial increase. On the other hand, an example of a patient whose initial prostate volume was 42 cm^3^, with a 0.7 mm increase in MRD, is shown in Figure 1. After the derived 14.7% volume increase the prostate volume would be 48.2 cm^3^. In line with Kim *et al.* [16], it is our conviction that such small expansions can be considered clinically irrelevant, despite the mean volume increase throughout the treatment being statistically significant, due to the limited effect on target coverage.

In this study, isotropic expansion of the prostate was assumed, although spatial anisotropy of the oedema has been reported, with average expansions of ∼ 10% in the D−V and C-C directions and ∼ 0% in the L−R direction [8]. In this study, the C-C direction was excluded, and the D-V and L-R directions were assumed equal. Furthermore, in contrast to Kim *et al.*, we did not consider the expansion in several planes separately to establish whether or not the prostate expanded isotropically at the base, middle and apex section of the prostate. They measured a small but not statistically significant increase of degree of volume change from apex to base.

Other studies reporting prostate volume changes for temporary brachytherapy treatments are listed in Table 2. Kiffer *et al.* [13] studied changes in implant geometry between the first and last HDR fraction. By estimating the cylindrical volume change from D-V and L-R implant coordinates, they found no change in volume in this period, but only considered 4 patients. Cury *et al.* [14] analysed prostate volumes of a larger patient group (31 patients) one week after implantation. They observed a large spread in volumetric changes. However, the determination of these volumes could be impaired by the relatively large slice thickness in combination with the delineation uncertainty on CT. In a prospective study on HDR monotherapy Martinez *et al.* reported that the volume increase took place during or shortly after needle placement [12]. During the subsequent 32–36 hours very little change in prostate volume occurred, resulting in a mean D_90%_ decrease of 4%. In our study we used CT instead of axial ultrasound planes and catheter positions instead of delineations. No CT was made prior to implantation, but the evaluated time span was longer.

**Table 2:** **Overview of studies performing volume measurements during temporary prostate brachytherapy**

**Author**	**Volume change**	**Range volume change**	**Imaging modality**	**Method volume assessment**	**# of patients**	**Treatment**	**# of catheters**	**Scans (h)**
Martinez et al.* [12]	+20% and +2.7%*	−13.7% − +17.3%*	TRUS, 5 mm slices	TRUS-based axial contours	23	4 HDR fractions	NA	−1.5, 0, 32–36*
Kiffer *et al.* [13]	+0.6%	−4% − +4%	CT, 5 mm slices	Needle positions in axial plane	4	3-4 HDR fractions	17	0, 23.5-49.5
Cury *et al.* [14]	+3.4%	−14.2% − +23.8%	CT, 5 mm slices	CT-based axial contours	31	1 HDR fraction	17	0, 168
Kim *et al.* [16]	+7.8%	−12.8% − +16.8	CT, 3 mm slices	catheter positions in 3 axial planes	13	2 HDR fractions	14-18	0, 17-22
Dinkla et al.** [21]	+0% and +0.2%**	−14.7% − +5.2%**	CT, 2 mm slices	CT-based axial contours	31	24 PDR pulses	12	0, 24, 48**
This study**	+4.3% and +0.6%**	−9.3% − +15.6%**	CT, 2 mm slices	catheter positions in all planes	23	24 PDR pulses	12	0, 24, 48**

Scan times were reported as hours after the first scan, where 0 are the images captured for treatment planning.

*Two intervals were studied. Mean is shown for both intervals. Only the range of the second interval is shown, between 1.5 and 32–36 hours after implantation.

**Two intervals were studied. Mean is shown for both intervals. Only the range of the first interval is shown, between 1.5 and 24 hours after implantation.

A previous study on our data showed that the prostate volumes of the CT-based prostate delineations did not vary significantly (Table 2) [21]. The same subset of 23 patients as in the present study had a median change of +0.6% (range −7.8% to +3.2%) for CT1-CT2 and 0.0% (range, −7.6% to 9.2%) for CT1-CT3. No statistically significant correlation was found between the volume changes in that study and the volume changes derived from the MRDs. However, those PTVs were based on CT scans on which the visibility of the prostate boundaries is hampered. Furthermore, the delineations on the repeat CTs were supported by the delineation of the planning CT and the fiducial markers. As reported in the present paper, the distance between the markers can change up to 7–8 mm, making them less reliable as landmarks.

The mean inter-marker distances did not change substantially between subsequent scans. A systematic increase of 1.1%-2.8% was measured, corresponding to an increase of 0.6 mm. Even though this could indicate a trend of prostate expansion, the largest marker displacements were observed in the C-C direction. Marker motion in that direction can more likely be explained by instability of the markers. The markers are implanted through a needle, creating the possibility to move along the track left by the needle, before fixating in the gland. Changes in inter-marker distance were statistically significant for CT2-CT3 and CT1-CT3, whereas for the MRD the changes were statistically significant for CT1-CT2 and CT1-CT3. To calculate the MRD, catheter displacements of around 80 points in L-R and D-V direction were used. Inter-marker distances were based on absolute (3D) vectors between three marker pairs. The uncertainty in marker position has a much larger effect on this measurement. Besides the difficulty in defining the centre of the cylindrically shaped 5 mm markers (1 mm diameter), rotational motion could not be taken into account. Finally, it is unknown if positional changes whether the positional changes of the markers are truly caused by changes in prostate volume or whether another mechanism causes them to displace.

The average displacements of the dwell positions measured by catheter-based registration were small, especially in the L-R and D-V directions, where the mean shift was 0 mm with a maximum SD of 0.8 mm. We conclude that the catheter configuration remained stable throughout the treatment. The C-C direction showed the largest displacements, with a mean shift of 0 mm ±1.9 mm in the first 24 hours. In the catheter-based registration, the two reconstructed catheter sets were aligned as good as possible, thereby possibly compensating for total implant displacement. This also explained why the measured catheters displacement between CT1-CT3 were slightly smaller than the sum of CT1-CT2 and CT2-CT3.

With an average number of 80 dwell positions, the catheter-based registrations are relatively stable. However, since that method is not able to detect entire implant shifts, a marker-based registration was performed as well. The marker-based registration revealed slightly larger displacements than the catheter-based registration, also in the C-C direction. This was partly due to inaccuracy in the marker coordinates (1 mm SD). The marker-based registration of the phantom images also resulted in slightly larger shifts than with the catheter-based registration. Furthermore, the changes in inter-marker distance revealed instability of the markers, which also decreases the accuracy of marker-based registration. Despite the data being slightly more skewed to caudal displacements, interpretation of the direction of catheter displacement was therefore somewhat hampered. Note that craniocaudal displacements are unrelated to changes in the prostate volume itself, but could be caused by tissue oedema between the prostatic apex and the perineum [29]. Peri-prostatic oedema and oedema in the prostate lateral to the catheters could not be evaluated with our methods. But since the catheters were placed on the periphery of the prostate (Figure 1), we expect to cover the entire prostate volume by analysing volume changes within the catheter space.

Damore *et al*. studied catheter and marker displacements in the C-C direction on pelvic radiographs [30]. They also note instability of the markers, with a mean displacement of 3.6 mm. Because of the use of a template, their catheters shifted as a whole. Catheter displacements between the first two fractions relative to the pubic symphysis (mean 8.3 mm) were larger than relative to the markers (mean 6.8 mm), which was explained by part of the motion of the markers and catheters being in the same direction. The displacement of their needles was larger than ours, due to the use of a template and smooth stainless steel needles, as compared to our self-anchoring needles. However, they reported that the displacement was partly overestimated by the use of anterior films that magnify the displacement. Taking two CT-scans spaced 20 hours apart, Kim *et al*. found an average magnitude of caudal catheter displacement of 2.7 mm (range −6.0 to 13.5 mm) after bone registration and 5.4 mm (range −3.75 to 18.0 mm) for their marker-based method [31]. They did not report on the uncertainty of the marker-based method, but considered the marker-based method to be more accurate as the prostate can move relative to the bony anatomy. Foster *et al.* found comparable craniocaudal displacement (mean 5.1 mm) after marker-based registration between day 1 and day 2 [32].

A previous study on the feasibility of our self-anchoring catheters showed that the craniocaudal displacement of the tips was minimal [26]: a mean absolute tip displacement of 1.0 mm (range 0–6 mm) was measured after marker-based landmark registration of the two scans. Only the C-C direction was taken into account. In the current analysis the complete implant configuration in all three directions was evaluated in order to study volumetric changes of the prostate; however, the majority of the displacement could still be attributed to displacements in the C-C direction. The mean absolute displacements were slightly larger than the previously measured displacements [26]. Compared to other studies, the craniocaudal displacements of the self-anchoring catheters were modest [30-32].

Although we consider our method to be accurate, the accuracy of our method was limited by the accuracy in catheter reconstruction. The reported accuracy of catheter reconstruction is 1 mm ± 0.5 mm (1 SD) [33], which was larger than the mean differences between dwell positions after catheter-based registration. Reconstruction was performed manually on the axial slices and checked on the reconstructed sagittal view. The diameter of the catheters was 2 mm and CT pixel size was 0.94 × 0.94 mm^2^ with a slice thickness of 2 mm. We use plastic, flexible self-anchoring catheters with a diameter of 2 mm. The catheters are usually reconstructed by defining 4 points on axial planes through the catheters. Others focussed on the precision of source positioning inside the catheter, resulting in a standard deviation for source-positioning offset of ±1.1 mm for PDR afterloaders [34], adding another small uncertainty to the dose delivery.

In this study we have not studied the effect of changes in implant geometry on dosimetry. Our results showed no significant motion in L-R or D-V direction larger than the reconstruction uncertainty. A study by Pantelis *et al*. showed that uncertainties in catheter reconstruction up to 2 mm and uncertainties in source position of the order of 1.5 mm along the catheter have no significant impact (less than 3%) on the dose-volume histogram (DVH) [35]. We can therefore expect that the dwell position shifts found in this study also have minor effect on the dose distribution. Pantelis *et al*. also concluded that a 4 mm caudal displacement of all catheters resulted in a D_90%_ decrease of −24%, showing that if the entire implant shifts the dose coverage is compromised. Considering that the base of the prostate is often delineated too small on CT [22], caudal displacements of the catheters are of added concern when it comes to sufficient dose coverage in the base of the prostate. However, in an earlier study [21] we demonstrated that in our patient group the mean deviation in prostate V_100%_ was −3%, making an entire implant shift unlikely when self-anchoring catheters are used.

## Conclusions

Applying four different methods to investigate catheter geometry changes inside the prostate revealed that both in the first 24 hours and in the second 24 hours of the temporary prostate brachytherapy treatment no clinically relevant volume changes occur that can have significant impact on dose delivery. Apart from some craniocaudal displacement, the catheters remain stable during these 2 days of treatment, confirming the safety of this technique.

## Abbreviations

C-C: Craniocaudal
CT: Computed tomography
D-V: Dorsoventral
DVH: Dose-volume histogram
D_90%_: Minimal dose achieved in 90% of the volume
EBRT: External beam radiotherapy
HDR: High-dose rate
ICP: Iterative closest point
LDR: Low-dose rate
L-R: Left right
MRD: Mean radial distance
PDR: Pulsed-dose rate
RMS: Root mean square
SD: Standard deviation
TRUS: Transrectal ultrasound
V_100%_: Percentage of volume receiving at least 100% of the prescribed dose

## References

[1] Hoskin PJ, Rojas AM, Bownes PJ, Lowe GJ, Ostler PJ, Bryant L (2012). Randomised trial of external beam radiotherapy alone or combined with high-dose-rate brachytherapy boost for localised prostate cancer. Radiother Oncol.

[2] Pieters BR, Geijsen ED, Koedooder C, Blank LE, Rezaie E, van der Grient JN, de Reijke TM, Koning CC (2011). Treatment results of PDR brachytherapy combined with external beam radiotherapy in 106 patients with intermediate- to high-risk prostate cancer. Int J Radiat Oncol Biol Phys.

[3] Lettmaier S, Lotter M, Kreppner S, Strnad A, Fietkau R, Strnad V (2012). Long term results of a prospective dose escalation phase-II trial: interstitial pulsed-dose-rate brachytherapy as boost for intermediate- and high-risk prostate cancer. Radiother Oncol.

[4] Pieters BR, Rezaie E, Geijsen ED, Koedooder C, van der Grient JN, Blank LE, de Reijke TM, Koning CC (2011). Development of late toxicity and international prostate symptom score resolution after external-beam radiotherapy combined with pulsed dose rate brachytherapy for prostate cancer. Int J Radiat Oncol Biol Phys.

[5] Kirisits C, Rivard MJ, Baltas D, Ballester F, De Brabandere M, van der Laarse R, Niatsetski Y, Papagiannis P, Hellebust TP, Perez-Calatayud J, Tanderup K, Venselaar JL, Siebert FA (2013). Review of clinical brachytherapy uncertainties: analysis guidelines of GEC-ESTRO and the AAPM. Radiother Oncol.

[6] Pinkawa M, Asadpour B, Gagel B, Piroth MD, Borchers H, Jakse G, Eble MJ (2007). Evaluation of source displacement and dose–volume changes after permanent prostate brachytherapy with stranded seeds. Radiother Oncol.

[7] Tanaka O, Hayashi S, Matsuo M, Nakano M, Uno H, Ohtakara K, Miyoshi T, Deguchi T, Hiroaki H (2007). Effect of edema on postimplant dosimetry in prostate brachytherapy using CT/MRI fusion. Int J Radiat Oncol Biol Phys.

[8] Sloboda RS, Usmani N, Pedersen J, Murtha A, Pervez N, Yee D (2010). Time course of prostatic edema post permanent seed implant determined by magnetic resonance imaging. Brachytherapy.

[9] Waterman FM, Yue N, Corn BW, Dicker AP (1998). Edema associated with I-125 or Pd-103 prostate brachytherapy and its impact on post-implant dosimetry: an analysis based on serial CT acquisition. Int J Radiat Oncol Biol Phys.

[10] Ghilezan M (2012). Role of high dose rate brachytherapy in the treatment of prostate cancer. Cancer Radiother.

[11] Vicini F, Vargas C, Gustafson G, Edmundson G, Martinez A (2003). High dose rate brachytherapy in the treatment of prostate cancer. World J Urol.

[12] Martinez AA, Pataki I, Edmundson G, Sebastian E, Brabbins D, Gustafson G (2001). Phase II prospective study of the use of conformal high-dose-rate brachytherapy as monotherapy for the treatment of favorable stage prostate cancer: a feasibility report. Int J Radiat Oncol Biol Phys.

[13] Kiffer JD, Schumer WA, Mantle CA, McKenzie BJ, Feigen M, Quong GG (2003). Impact of oedema on implant geometry and dosimetry for temporary high dose rate brachytherapy of the prostate. Australas Radiol.

[14] Cury FL, Duclos M, Aprikian A, Patrocinio H, Souhami L (2010). Prostate gland edema after single-fraction high-dose rate brachytherapy before external beam radiation therapy. Brachytherapy.

[15] Smith WL, Lewis C, Bauman G, Rodrigues G, D’Souza D, Ash R, Ho D, Venkatesan V, Downey D, Fenster A (2007). Prostate volume contouring: a 3D analysis of segmentation using 3DTRUS, CT, and MR. Int J Radiat Oncol Biol Phys.

[16] Kim Y, Hsu IC, Lessard E, Vujic J, Pouliot J (2004). Dosimetric impact of prostate volume change between CT-based HDR brachytherapy fractions. Int J Radiat Oncol Biol Phys.

[17] Kovalchuk N, Furutani KM, Macdonald OK, Pisansky TM (2011). Dosimetric effect of interfractional needle displacement in prostate high-dose-rate brachytherapy. Brachytherapy.

[18] Huang Y, Miller B, Doemer A, Babij D, Kumar S, Frontera R, Nurushev T, Chetty IJ, Aref I (2013). Online correction of catheter movement using CT in high-dose-rate prostate brachytherapy. Brachytherapy.

[19] Whitaker M, Hruby G, Lovett A, Patanjali N (2011). Prostate HDR brachytherapy catheter displacement between planning and treatment delivery. Radiother Oncol.

[20] Milickovic N, Mavroidis P, Tselis N, Nikolova I, Katsilieri Z, Kefala V, Zamboglou N, Baltas D (2011). 4D analysis of influence of patient movement and anatomy alteration on the quality of 3D U/S-based prostate HDR brachytherapy treatment delivery. Med Phys.

[21] Dinkla AM, Pieters BR, Koedooder C, Meijnen P, van Wieringen N, van der Laarse R, van der Grient JN, Rasch CR, Bel A (2013). Deviations from the planned dose during 48 hours of stepping source prostate brachytherapy caused by anatomical variations. Radiother Oncol.

[22] Dinkla AM, Pieters BR, Koedooder C, van Wieringen N, van der Laarse R, van der Grient JN, Rasch CR, Koning CC, Bel A (2013). Improved tumour control probability with MRI-based prostate brachytherapy treatment planning. Acta Oncol.

[23] Yamada Y, Rogers L, Demanes DJ, Morton G, Prestidge BR, Pouliot J, Cohen GN, Zaider M, Ghilezan M, Hsu IC (2012). American Brachytherapy Society consensus guidelines for high-dose-rate prostate brachytherapy. Brachytherapy.

[24] Kovacs G, Potter R, Loch T, Hammer J, Kolkman-Deurloo IK, de la Rosette JJ, Bertermann H (2005). GEC/ESTRO-EAU recommendations on temporary brachytherapy using stepping sources for localised prostate cancer. Radiother Oncol.

[25] Hoskin PJ, Colombo A, Henry A, Niehoff P, Paulsen HT, Siebert FA, Kovacs G (2013). GEC/ESTRO recommendations on high dose rate afterloading brachytherapy for localised prostate cancer: an update. Radiother Oncol.

[26] Pieters BR, van der Grient JN, Blank LE, Koedooder C, Hulshof MC, de Reijke TM (2006). Minimal displacement of novel self-anchoring catheters suitable for temporary prostate implants. Radiother Oncol.

[27] Besl PJ, McKay N (1992). A method for registration of 3-D shapes. IEEE PAMI.

[28] Horn BKP (1987). Closed-form solution of absolute orientation using unit quaternions. J Opt Soc Am.

[29] Hoskin PJ, Bownes PJ, Ostler P, Walker K, Bryant L (2003). High dose rate afterloading brachytherapy for prostate cancer: catheter and gland movement between fractions. Radiother Oncol.

[30] Damore SJ, Syed AM, Puthawala AA (2000). Needle displacement during HDR brachytherapy in the treatment of prostate cancer. Int J Radiat Oncol Biol Phys.

[31] Kim Y, Hsu IC, Pouliot J (2007). Measurement of craniocaudal catheter displacement between fractions in computed tomography-based high dose rate brachytherapy of prostate cancer. J Appl Clin Med Phys.

[32] Foster W, Cunha JA, Hsu IC, Weinberg V, Krishnamurthy D, Pouliot J (2011). Dosimetric impact of interfraction catheter movement in high-dose rate prostate brachytherapy. Int J Radiat Oncol Biol Phys.

[33] Tsalpatouros A, Baltas D, Kolotas C, van der Laarse R, Koutsouris D, Uzunoglu NK, Zamboglou N (1997). CT-based software for 3-D localization and reconstruction in stepping source brachytherapy. IEEE Trans Inf Technol Biomed.

[34] Elfrink RJ, Kolkman-Deurloo IK, van Kleffens HJ, Rijnders A, Schaeken B, Aalbers TH, Dries WJ, Venselaar JL (2001). Determination of the accuracy of implant reconstruction and dose delivery in brachytherapy in The Netherlands and Belgium. Radiother Oncol.

[35] Pantelis E, Papagiannis P, Anagnostopoulos G, Baltas D, Karaiskos P, Sandilos P, Sakelliou L (2004). Evaluation of a TG-43 compliant analytical dosimetry model in clinical 192Ir HDR brachytherapy treatment planning and assessment of the significance of source position and catheter reconstruction uncertainties. Phys Med Biol.

